# Tumor energy metabolism: implications for therapeutic targets

**DOI:** 10.1186/s43556-024-00229-4

**Published:** 2024-11-29

**Authors:** Youwu Hu, Wanqing Liu, WanDi Fang, Yudi Dong, Hong Zhang, Qing Luo

**Affiliations:** 1 The Public Experimental Center of Medicine, Affiliated Hospital of Zunyi Medical University, 149 Dalian Road, Huichuan District, Zunyi, Guizhou 563003 China; 2 Guizhou Provincial Key Laboratory of Cell Engineering, Affiliated Hospital of Zunyi Medical University, Zunyi, China; 3 Department of Pathology, Affiliated Hospital of Zunyi Medical University, Zunyi, China

**Keywords:** Tumor energy metabolism, Glycolysis, Mitochondrial phosphorylation, Therapeutic targets, Drug resistance, SWI/SNF complex

## Abstract

Tumor energy metabolism plays a crucial role in the occurrence, progression, and drug resistance of tumors. The study of tumor energy metabolism has gradually become an emerging field of tumor treatment. Recent studies have shown that epigenetic regulation is closely linked to tumor energy metabolism, influencing the metabolic remodeling and biological traits of tumor cells. This review focuses on the primary pathways of tumor energy metabolism and explores therapeutic strategies to target these pathways. It covers key areas such as glycolysis, the Warburg effect, mitochondrial function, oxidative phosphorylation, and the metabolic adaptability of tumors. Additionally, this article examines the role of the epigenetic regulator SWI/SNF complex in tumor metabolism, specifically its interactions with glucose, lipids, and amino acids. Summarizing therapeutic strategies aimed at these metabolic pathways, including inhibitors of glycolysis, mitochondrial-targeted drugs, exploitation of metabolic vulnerabilities, and recent developments related to SWI/SNF complexes as potential targets. The clinical significance, challenges, and future directions of tumor metabolism research are discussed, including strategies to overcome drug resistance, the potential of combination therapy, and the application of new technologies.

## Introduction

Tumor energy metabolism is the process by which tumor cells convert and utilize energy for growth and survival, involving the reprogramming of metabolic pathways. Tumor cells metabolize energy differently from normal cells by reprogramming various pathways, such as aerobic respiration, anaerobic fermentation, and fatty acid oxidation. The metabolic characteristics of tumors are closely related to their biological behavior. Tumor cells frequently show uncontrolled growth and proliferation, necessitating metabolic reprogramming to satisfy their increased energy and biosynthetic demands. This reprogramming also helps reduce oxidative stress, enabling tumor cells to adapt to varying energy requirements in different physiological and pathological conditions. For instance, normal cells generate energy via oxidative phosphorylation when oxygen is available, whereas tumor cells rely on glycolytic pathways for energy. Glycolysis produces energy at a faster rate and also produces a variety of intermediate metabolites that can be used to synthesize nucleotides and amino acids to support the rapid proliferation of cells [1]. Lactic acid generated by glycolysis can inhibit the effector functions of immune cells in the tumor microenvironment (TME), thereby facilitating tumor evasion and progression [2]. These metabolic changes support tumor cell survival in harsh conditions, facilitate tumor development, and offer potential targets for early diagnosis and targeted therapy.

Otto Warburg’s observations were the starting point for the study of cancer metabolism. He discovered the famous “Warburg effect” in the early twentieth century, in which tumor cells metabolize glucose to lactate even under aerobic conditions, and he believes that impaired respiration of cancer cells is a prerequisite for cells to become cancerous [3–5]. Over the next few decades, researchers tried to explain how the Waberg effect promotes tumor growth. Two key findings indicate that glycolysis and TCA cycle metabolites promote tumor growth. They do this by facilitating biosynthesis and acting as signaling molecules. One of the key findings is that ATP produced by glycolysis can support primary tumor growth after the electron transport chain (ETC) in cancer cells is disrupted [6]. The TCA circulating metabolite oxaloacetate has been shown to be required for primary and metastatic tumor growth [7, 8]. Another finding is that reactive oxygen species (ROS) produced by oxidative metabolism can support tumorigenesis [9]. And ROS metabolites can act as signaling molecules to promote tumor growth by controlling gene expression [10]. As research progressed, scientists began to explore how tumor cells alter metabolic pathways. It includes multiple metabolic pathways such as glycolysis, oxidative phosphorylation, and fatty acid metabolism. Signaling pathways such as MYC, AKT, mTOR and HIFs discovered in the 90 s of the twentieth century increase glycolysis through transcriptional regulation and phosphorylation.

Recently, the study of tumor energy metabolism has become one of the hot spots in tumor biology and treatment research [11–14]. A variety of metabolism-based antitumor drugs have shown good results in experiments. For example, small molecule inhibitors developed for the glycolytic pathway, such as the glucose transporter 1 (GLUT1) inhibitor Bay-876 and the hexokinase (HK2) inhibitor Benserazide (Benz) [15–17], as well as Ab3-810, a monoclonal antibody targeting glutamine transporter 5 (SLC1A5), and metformin, a drug targeting mitochondrial complex I, can activate AMPK, thereby inhibiting the growth, survival, and metastasis of various tumor cells such as breast cancer, liver cancer, and pancreatic cancer [18–20].

Despite these advances, translating these findings into effective clinical interventions remains challenging, particularly due to issues such as drug resistance stemming from the metabolic adaptations of tumors and variability in their responses to treatment. Previous studies have focused more on the metabolic pathways of cells and their contribution to energy production, and the energy metabolism of tumor cells is usually regarded as a relatively static process, and it is difficult to describe the dynamic change process of metabolism and the interaction between metabolites and tumors. Recent studies have focused on the dynamic properties of tumor energy metabolism, especially the bidirectional relationship between epigenetics and tumor metabolism [21]. Epigenetic modifications affect the metabolic state and biological behavior of tumors by regulating the expression of metabolism-related genes without altering the DNA sequence [22, 23]. Tumor metabolites regulate gene expression through epigenetic modifications. For example, metabolites such as lactate are not only a result of energy metabolism, but can also affect the growth and metastatic ability of tumor cells by regulating epigenetic states [24].

Therefore, understanding how tumor energy metabolism relates to epigenetic regulation can help develop new treatment strategies. This review starts with an introduction to key processes in tumor metabolism, including glycolysis and mitochondrial function, as well as tumor adaptation and plasticity. Specially, we focus on the epigenetic factor-SWI/SNF chromatin remodeling complex, which showed closely relationship to tumor metabolism but lack in-depth enough knowledge, in order to provide new ideas for the development of tumor metabolism therapy and targeted drugs. Finally, strategies targeting metabolic pathways was discussed, not only the latest clinical trails but also future research directions. This will provide innovative approaches to cancer treatment based on tumor energy metabolism.

## The main pathways of energy metabolism in tumors

Tumor cells require significant energy and materials for rapid growth, and their metabolism is regulated through aerobic glycolysis and mitochondrial oxidative phosphorylation. This adaptation helps cells thrive in the low-oxygen tumor microenvironment. It also provides the energy and materials needed for biological activities like tumor growth, invasion, and migration. Tumor metabolism is regulated at both the gene and protein levels. Additionally, genomic instability can alter metabolic pathways, impacting how cells produce and utilize energy. For instance, the elevated expression of the proto-oncogene MYC enhances the levels of key enzymes in both glycolysis and fatty acid synthesis, leading to increased energy resource production [25]. The activity of proteins, such as hexokinase (HK) and phosphofructokinase (PFK), which are key enzymes in the glycolytic pathway, directly affects the rate of glucose metabolism [26].

### Glycolysis and the Waburg effect

#### Transition of tumor cells to aerobic glycolysis

Glycolysis is an important process in cellular metabolism, and tumor cells often exhibit high levels of glycolysis known as the "Warburg effect" or "aerobic glycolysis". This process occurs in the cytoplasm and consists of two phases: the first stage converts glucose into pyruvate, NADH, and 2 ATP molecules, while the second phase involves fermentation, in which pyruvate is reduced and metabolized into organic acids or alcohols, such as lactic acid.

The special needs of tumor cells for energy metabolism are closely related to their ability to grow and metastasize. Aerobic glycolysis produces ATP more quickly and maintains high levels of glycolytic intermediates to support the anabolic reactions necessary for synthesizing macromolecules like DNA, RNA, proteins, and lipids, thus facilitating rapid growth and proliferation [27]. Lactic acid produced by glycolysis leads to the acidification of the tumor microenvironment, which helps inhibit the immune response and promotes the proliferation and invasion of tumor cells [28, 29]. Glycolysis generates significantly fewer reactive oxygen species (ROS) than oxidative phosphorylation, which protects tumor cells from oxidative stress and contributes to their resistance to apoptosis [30]. Aerobic glycolysis does not require mitochondria to maintain redox balance, as NADH produced by glycolysis is oxidized back to NAD + when pyruvate is converted to lactic acid, allowing NAD + to continue supporting the glycolytic cycle [31].

#### The role of key enzymes and their regulatory mechanisms

Glycolysis involves 10 enzymatic steps. The three key enzymes that catalyze irreversible reactions—HK, phosphofructokinase-1 (PFK1), and pyruvate kinase (PK)—along with glucose transporter (GLUT), PFK, glyceraldehyde-3-phosphate dehydrogenase (GAPDH), pyruvate dehydrogenase (PDH), and lactate dehydrogenase (LDH). Studies have found that overexpression of these enzymes can enhance the metabolic activity of tumor cells, which in turn promotes their proliferation and survival.

HK promotes the growth and proliferation of cells primarily by converting glucose to glucose-6-phosphate. Different subtypes of HK (e.g., HK1, HK2, HK3, and HK4) exhibit different expression patterns in different types of tumors. The overexpression of HK2 in a variety of tumors is closely related to the aggressiveness and metastasis of tumors [32].

PKM2 is responsible for the conversion of phosphoenolpyruvate to pyruvate and, in the process, ATP is generated. It is usually highly expressed in tumor cells and is present in both active tetrameric and inactive dimer states [33]. In the dimer state, PKM2 has low catalytic activity, which allows cells to accumulate glycolytic intermediates that can not only enter the glycolytic pathway, but also be converted into substrates for other metabolic pathways, thus providing cells with the necessary biosynthetic precursors and energy requirements to promote rapid proliferation and growth of tumor cells [34].

PFK1 is the primary regulatory point in response to cellular energy levels and allosteric effectors, responsible for the conversion of fructose-6-phosphate to fructose-1,6-bisphosphate. PFK activity is regulated by a variety of metabolites, including ATP, ADP, and fructose-2,6-bisphosphate. In tumor cells, PFK expression and activity are often significantly increased, promoting enhanced glycolysis [35]. The overexpression of PFK is closely related to cell proliferation, migration and survival [36].

LDH is primarily responsible for the reduction of pyruvate to lactate. The expression level of LDH is closely related to the aggressiveness and prognosis of tumors. Especially in malignant tumors such as pancreatic cancer and lung cancer, the high expression of LDH-A is positively correlated with the degree of malignancy of the tumor. LDH-A affects cancer cell metabolism by controlling the NADH/NAD ratio, and elevated LDH-A levels lead to tumor metastasis by promoting EMT [37]. The production of lactate during glycolysis and the release of tumor microenvironment (TME) acidosis are major factors, which lead to high tumor invasion, migration, and immune cell suppression. Inhibition of LDH can effectively reduce the glucose uptake and proliferation of tumor cells, and at the same time improve the function of tumor infiltrating T cells, thereby enhancing anti-tumor immunity [38].

Many studies have shown that key enzymes are regulated by transcription, metabolite feedback, and cell signaling pathways. Expression of almost all glycolytic enzymes are upregulated by transcription factors c-MYC (MYC) and Hypoxia-inducible factor-1 (HIF-1). Including hexokinase II (HK II), phosphofructokinase (PFK), Enolase 1 (ENO1), pyruvate dehydrogenase (PDH), pyruvate kinase (PKM2) and lactate dehydrogenase (LDHA). c-MYC and HIF-1 also are the driver of the metabolic transition from oxidation to glycolysis. They cooperation in activating HK2 further enhances the retention of glucose for its glycolytic conversion to lactate, thereby contributing to the Warburg effect [39]. After c-MYC is reduced, the expression of HK2, PFKM, PKM2 and LDHA in colon cancer cells is reduced, glycolysis is inhibited, and intracellular ATP is reduced, resulting in endoplasmic reticulum stress and immunogenic cell death in colorectal cancer cells [40]. The activity of PFK-1 is regulated by a variety of metabolites. For example, PFKFB3 is a potent PFK-1 activator. Studies have shown that PFKFB3 is upregulated in a variety of cancers, and PFKFB3 overexpression can enhance glycolysis in ovarian cancer cells, increase lactate production, and reduce cellular oxygen consumption, supporting tumor growth, chemoresistance, metastasis and stemness in ovarian cancer [41]. Activation of cell signaling pathways, such as the PI3K-AKT-mTOR pathway, increases the amount of GLUT1 and GLUT12 on the cell membrane, increases glucose uptake by tumor cells, and produces the required ATP through aerobic glycolysis for proliferation, migration, and invasion. As a consequence of the aerobic glycolysis, extracellular acidification also provides GLUT12 with the electrochemical gradient of H + to cotransport and accumulate glucose inside the cell, further feeding the aerobic glycolysis [42].

### Mitochondrial function and the role of oxidative phosphorylation in tumor metabolism

Mitochondria are the main energy production centers in cells, and tumor cells often adapt to their living environment by changing the function and metabolic pathways of mitochondria, thereby promoting the growth and metastasis of tumors. For example, tumor cells enhance their proliferation and survival by increasing their uptake of pyruvate and glutamate. This process promotes mitochondrial energy production [43]. ATP synthesis and metabolite production in mitochondria are crucial for the viability of tumor cells. If mitochondria malfunction, it can lead to insufficient energy supply, negatively impacting tumor cell proliferation and metastasis [44]. Understanding the function of mitochondria and their role in tumor metabolism is important for the development of new cancer treatment strategies.

Oxphosphorylation (OXPHOS) is a key function of mitochondria that primarily takes place in the inner mitochondrial membrane. This process transfers electrons from nutrients like glucose and fatty acids to oxygen via electron transport chains. This reaction forms water and releases energy, which is then used to synthesize ATP. Additionally, mitochondria regulate metabolic pathways including glycolysis, fatty acid oxidation (FAO), and amino acid metabolism [45]. Within the mitochondria, pyruvate and fatty acids are converted to acetyl-CoA, which then enters the tricarboxylic acid cycle (TCA cycle), where NADH and FADH2 are further generated. These reduced equivalents are eventually converted to ATP via the electron transport chain (ETC). The electron transport chain consists of several complexes, including complex I (NADH dehydrogenase), complex II (succinate dehydrogenase), complex III (cytochrome bc1 complex), and complex IV (cytochrome c oxidase). The individual proteins in the complex ensure efficient transport of electrons through their specific conformational changes and binding capacity. Research indicates that dysfunction in the electron transport chain is closely linked to various metabolic diseases and the aging process [46, 47].

Oxygen phosphorylation (OXPHOS) is a key function of mitochondria and occurs primarily in the inner mitochondrial membrane. This process transfers electrons from nutrients such as glucose and fatty acids to oxygen through the electron transport chain. This reaction forms water and releases energy, which is then used to synthesize ATP. In ovarian cancer cells, higher OXPHOS activity is linked to increased tumor cell growth [48]. Estrogen receptor-positive breast cancer cells exhibit increased OXPHOS, which is associated with tumor cell proliferation and viability [49]. OXPHOS influences immune cell function and tumor immune escape mechanisms by regulating the metabolic states within the tumor microenvironment [50]. Mitochondrial dysfunction in specific cancer subtypes is closely related to their clinical features. For instance, glioma stem cells demonstrate higher mitochondrial activity and expression of OXPHOS proteins compared to isogenetically differentiated glioma cells [51]. Cancer stem cells in cholangiocarcinoma rely on mitochondrial oxidative metabolism and peroxisome proliferator-activated receptor γ coactivator (PGC-1α) to maintain their characteristics [52]. Breast cancer cell subtypes exhibit different metabolic phenotypes, and these metabolic signatures are directly related to their clinical classification [53]. Triple-negative breast cancer is highly dependent on OXPHOS, especially when chemotherapy is suppressed, and inhibition of OXPHOS can enhance the effect of chemotherapy [54]. Osteogenic differentiation in breast cancer cells activates both the classical TGF-β/Smad signaling pathway and the non-canonical MAPK pathway. This process enhances OXPHOS, promoting the epithelial-mesenchymal transition (EMT). The EMT phenotype can be reversed by inhibiting OXPHOS with rotenone [55]. Estrogen upregulation in breast cancer cells increases the content of mitochondrial biogenesis (PGC1-α, TFAM) and transcription factors associated with OXPHOS (OCTN2, ND1, COX-IV) [56]. In gastric cancer, patients with the microsatellite instability (MSI) subtype have been found to be positively correlated with the activation of oxidative stress-related pathways [57].

Mitochondria also play a role in regulating glycolysis, fatty acid oxidation (FAO), and amino acid metabolism. In the mitochondrial matrix, pyruvate and fatty acids convert to acetyl-CoA. This acetyl-CoA then enters the tricarboxylic acid cycle (TCA cycle), where it is oxidized to produce NADH and FADH2. Glutamine is another important source of energy for tumors, participating in the TCA cycle to produce energy [58]. Without glucose, tumor cells sustain growth and survival by increasing fatty acid uptake and oxidation [59].

**Fig. 1:**
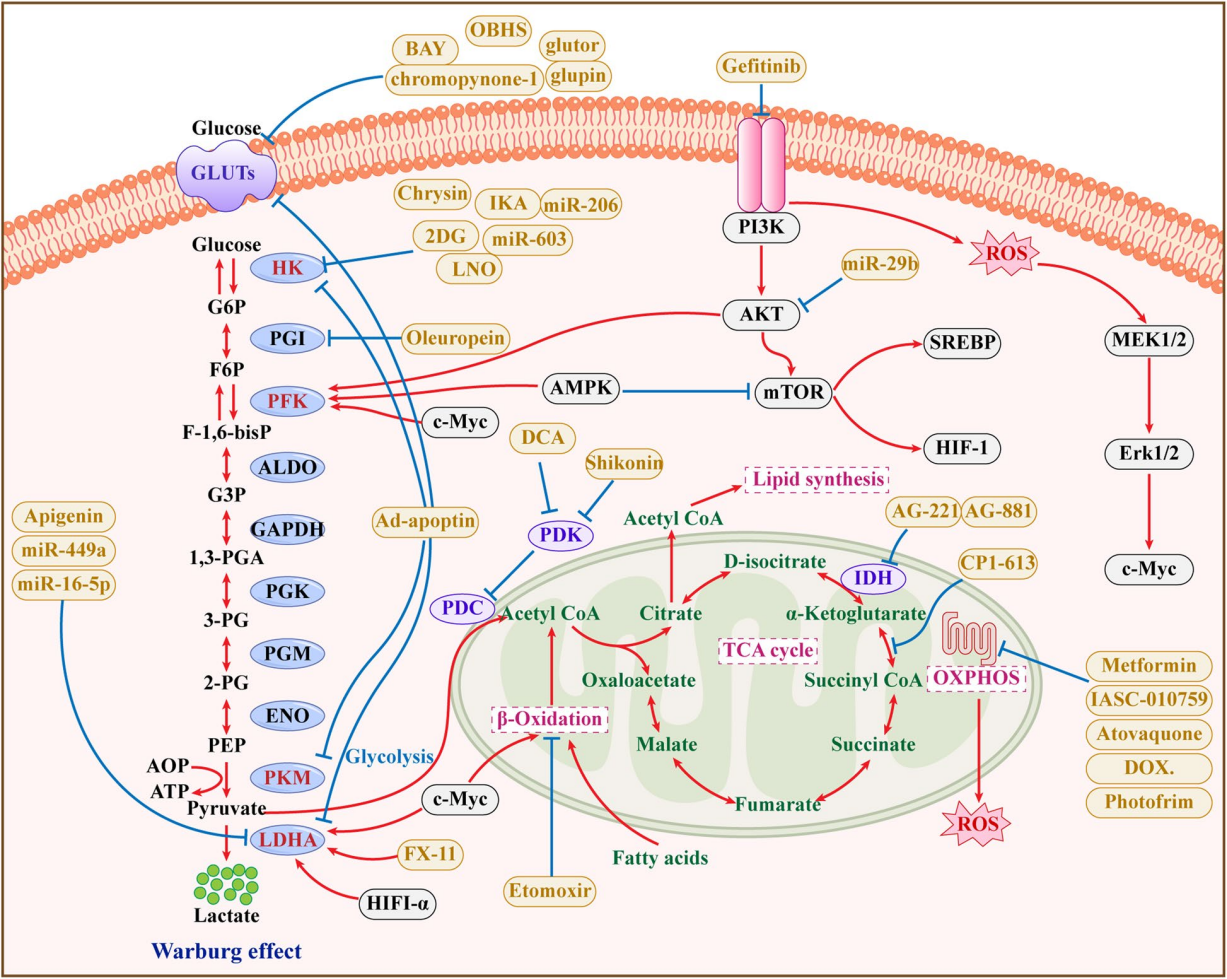
Inhibitors that target mitochondrial metabolism and Warburg effect The blue circles in the figure are the enzymes in the glycolysis and mitochondrial metabolism pathways, the yellow circles are the targeted inhibitors, and the white circles are the relevant regulatory factors

There is a complex interaction between glycolysis and mitochondrial oxidative phosphorylation, and their mutual regulation is essential for maintaining cellular energy balance and metabolic fitness. The interaction between glycolysis and oxidative phosphorylation is primarily evident in the flow of metabolites and energy demands. Under hypoxia or increased energy demands, glycolysis rates rise significantly to quickly provide energy. In contrast, when oxygen is plentiful, mitochondrial oxidative phosphorylation serves as the primary energy source. Metabolite feedback regulation is crucial for the mutual regulation of glycolysis and oxidative phosphorylation. The accumulation of lactate, a glycolytic byproduct, can further influence glycolysis rates by affecting pH and intracellular signaling pathways. At the same time, metabolites such as ATP and NADH produced by mitochondria can inhibit the activity of glycolysis-related enzymes through a negative feedback mechanism to maintain intracellular energy homeostasis [60, 61]. Activating signaling pathways like AMPK promotes glycolysis while inhibiting oxidative phosphorylation, leading to increased ATP production [62]. Warburg posits that 'aerobic glycolysis' in tumor cells indicates mitochondrial dysfunction. The contemporary view suggests that when mitochondria are overworked, glycolysis produces NADH faster than NAD can be regenerated, leading to saturated aerobic glycolysis. Consequently, tumor cells increase glycolysis for more energy, resulting in lactic acid production [63]. Mitochondrial dysfunction is closely related to the improvement of tumor drug resistance and malignancy, and targeting oxidative phosphorylation and the electron transport chain can effectively reduce the energy metabolism of tumor cells, thereby inhibiting their proliferation and metastasis [64] ( Fig. 1).

### Tumor metabolic plasticity is key to survival and drug resistance mechanisms

Tumor metabolic plasticity refers to the ability of tumor cells to flexibly adjust their metabolic pathways to adapt to survival and proliferation under different microenvironments and metabolic needs. This characteristic not only plays an important role in the occurrence and development of tumors, but also brings challenges to the treatment of tumors. Metabolic reprogramming involves activating transcription factors, abnormal signaling pathways, and altering the expression of metabolic enzymes. For instance, HIF-1α is activated in hypoxic conditions, increasing the expression of LDHA and enhancing glycolysis in tumor cells, which boosts their energy production and promotes proliferation and survival [65]. The increased expression and half-life of c-Myc protein can activate the β-TrCP/c-Myc/HK2 pathway, enhance glycolysis in colorectal cancer, significantly increase intracellular lactate, and promote the progression of colorectal cancer [66]. Additionally, tumor cells can be reprogrammed for rapid proliferation by modifying their fatty acid, amino acid, and nucleotide metabolism.

Cancer cells enhance their proliferation and survival by increasing fatty acid uptake and synthesis. They can synthesize up to 95% of saturated and monounsaturated fatty acids de novo. These fatty acids serve not only as energy sources but also play crucial roles in signal transduction and regulating cell functions. High levels of FASN are linked to a more aggressive tumor phenotype. Knocking out key enzymes related to fatty acid synthesis, such as FASN, ACC, and ACLY, can reduce cell proliferation and induce apoptosis in tumor cells [67–71]. Glutamate and leucine are highly utilized in cancer cells, and glutamine not only meets the energy needs of tumor cells but also provides carbon and nitrogen sources for macromolecular biosynthesis. For example, pancreatic cancer cells rely on glutamine metabolism to survive in a hypoxic environment [72, 73]. Colorectal cancer cells proliferate rapidly by remodeling the glutamine metabolic pathway [18]. Pancreatic cancer cells use the non-canonical KRAS-induced glutamine pathway to maintain redox homeostasis, supporting cell growth by increasing the nicotinamide adenine dinucleotide phosphate (NADPH/NADP +) ratio [74].

During cancer therapy, cancer cells often resist drug effects through metabolic reprogramming. For example, bladder cancer cells, when treated with cisplatin chemotherapy, rapidly shift to a metabolic pattern dependent on oxidative phosphorylation, thereby evading drug inhibition [75]. Pancreatic cancer's resistance to conventional treatment is partly due to the reprogramming of multiple metabolic pathways by the mutant KRAS gene. This mutation up-regulates glycolytic flux and enhances both the hexosamine biosynthesis pathway and the pentose phosphate pathway by increasing the expression of glycolytic enzymes such as GLUT1, HK 1/2, PFK, and LDHA. Researchers remodel glutamine metabolism in pancreatic cancer by increasing the expression of aspartate aminotransferase (GOT1) and decreasing glutamate dehydrogenase (GLUD1), thereby increasing flux through GOT1-dependent pathways. Additionally, researchers enhance lipid metabolism in pancreatic cancer cells by increasing the expression of carnitine palmitoyltransferase 1a (CPT1a), acyl-CoA oxidase (AOX), and peroxisome proliferator-activated receptor γ (PPARγ), which helps protect against metabolic stress caused by drugs. Furthermore, mutant KRAS expression can also increase NRF2 transcription, activate the ROS detoxification program, and help pancreatic cancer cells resist ROS [76, 77]. The TIAM2 gene, which is involved in lipid metabolism, increases lung adenocarcinoma (LAUD) cells' tolerance to osimertinib and enhances their motility [78]. ACLY promotes MITF-PGC1α axis transcription by increasing histone acetylation of the MITF locus, making melanoma resistant to MAPK inhibitors [79].

## Epigenetics and tumor energy metabolism

Epigenetic modifications regulate gene expression without changing the DNA sequence through mechanisms such as chromatin remodeling, DNA methylation, histone modifications, RNA modifications, and regulation of non-coding RNAs. These modifications can inhibit DNA repair mechanisms and increase genomic instability. It also impacts cell cycle regulation, allowing cells with unstable genomes to proliferate. This further exacerbates genomic damage and instability, which may significantly contribute to tumorigenesis and its progression [80, 81]. It also impacts cell cycle regulation, allowing cells with unstable genomes to proliferate. This further exacerbates genomic damage and instability, which may significantly contribute to tumorigenesis and its progression [82].

In addition, numerous studies have shown that tumor energy metabolism is closely related to epigenetic regulation. In the past few years, the study of m6A in tumor glycolysis has experienced an explosion of research, with numerous studies revealing the molecular mechanism by which m6A modification regulates tumor glycolysis and its key role in this metabolic process. m6A methylation affects the metabolic profile and biological behavior of tumors by affecting the stability of key enzyme genes [83]. Liu et al.’s study shows that m6A can modify the GLUT1 gene to enhance the stability of GLUT1 mRNA, thereby promoting the glycolysis and cell proliferation of Colorectal cancer [84]. Enolase (ENO1) promotes LAUD development in an m6A-dependent manner. Liu and colleagues found the combination of YTHDF1, a m6A “reader”, with methylated ENO1 mRNA leads to increased expression of ENO1 and promotes glycolysis of LAUD [85]. They also found m6A modification may enhance glycolytic capacity and development of LUAD by enhancing the stability of NPM1 to promote the expression of glycolytic enzymes (e.g., ENO1, HK2, LDHA, LDHB, and GLUT1) in LUAD [86]. Li et al. found that METTL3 promotes the expression of HK2 in an m6A-dependent manner, enhancing glycolysis of pancreatic ductal adenocarcinoma cells, thereby promoting their perineural invasion [87].

Currently, the most researched epigenetic mechanism in tumor metabolism is the regulation of non-coding RNAs. Including lncRNA、miRNA、circRNA and piRNA. LncRNAs are non-coding RNAs that are more than 200 nucleotides in length. LncRNA KCNQ1OT1 can stabilize HK2 in osteosarcoma, colorectal cancer, and ovarian cancer, enhancing aerobic glycolysis and promoting cancer progression [88, 89]. LncRNA LINC00671 is inversely correlated with LDHA levels, and inhibition of its expression can increase LDHA content in thyroid cancer cells and promote glycolysis, growth, and lung metastasis both in vitro and in vivo [90]. Circular RNA (circRNA) can specifically adsorb miRNA, and CircRNA microarray sequencing has shown that miR-487a carried by CircRNF20 in breast cancer can promote the binding of HIF-1α to the HK2 promoter, thereby enhancing the transcription and expression of HK2 and promoting the proliferation of breast cancer cells through the Warburg effect [91]. In gastric cancer, miR-3-515p carried by CircCUL3 can activate signal transducer and activator of transcription 3 (STAT3). This activation accelerates the transcription of HK2, ultimately triggering the Warburg effect and promoting gastric cancer development [91].

Another regulatory mechanism, chromatin remodeling, has emerged as an important factor in tumor metabolism. The mammalian SWI/SNF chromatin remodeling complex acts as an important epigenetic regulator, with genetic aberrations found in 25% of cancers [92, 93]. Numerous studies indicate a link between mutations in the SWI/SNF subunit and tumor metabolism. For instance, BRG1 can bind to the promoters of key fatty acid synthesis enzymes to regulate their transcription, affecting the expression of these key enzymes, such as fatty acid synthase, ATP citrate lyase, and acetyl-CoA carboxylase. This regulation impacts fatty acid synthesis in breast cancer cells, subsequently influencing their proliferation. Notably, BRG1’s role in regulating proliferation through fatty acid metabolism is unique to breast cancer [94, 95]. Drug development targeting SWI/SNF complexes, including small molecule inhibitors and immunotherapy strategies, has shown promising results in clinical trials, with patients responding well to these targeted therapies [96, 97]. Therefore, exploring the relationship between SWI/SNF complexes and tumor metabolism may provide important targets and ideas for new therapeutic strategies.

## The basic structure and function of the SWI/SNF complex

The SWI/SNF chromatin remodeling complex is a heterogeneous collection of related protein complexes required for gene regulation and genome integrity. It is an ATP-dependent chromatin reconstructor that regulates the accessibility of transcription factors to DNA by changing the assembly of nucleosomes, and reshapes the structure of chromatin by using the energy generated by ATP hydrolysis [98]. SWI/SNF complex is involved in the processes of cell mitosis, DNA replication, DNA damage repair, etc., and plays a role in cell differentiation and maintenance of cell state [99]. ARID1A subunit mutations are present in more than 50% of ovarian clear cell carcinomas and 40% of endometrioid carcinomas [100–102]. Therefore, its potential as a tumor biomarker is of increasing concern. Specific subunits of the SWI/SNF complex, such as ARID1A and SMARCA4 expression levels, mutation status, and copy number variation, can be used as diagnostic and prognostic markers for tumors. For example, ARID1A deletion is considered a characteristic hallmark of clear cell carcinoma of the ovary and is closely related to patient prognosis [103].

**Fig. 2:**
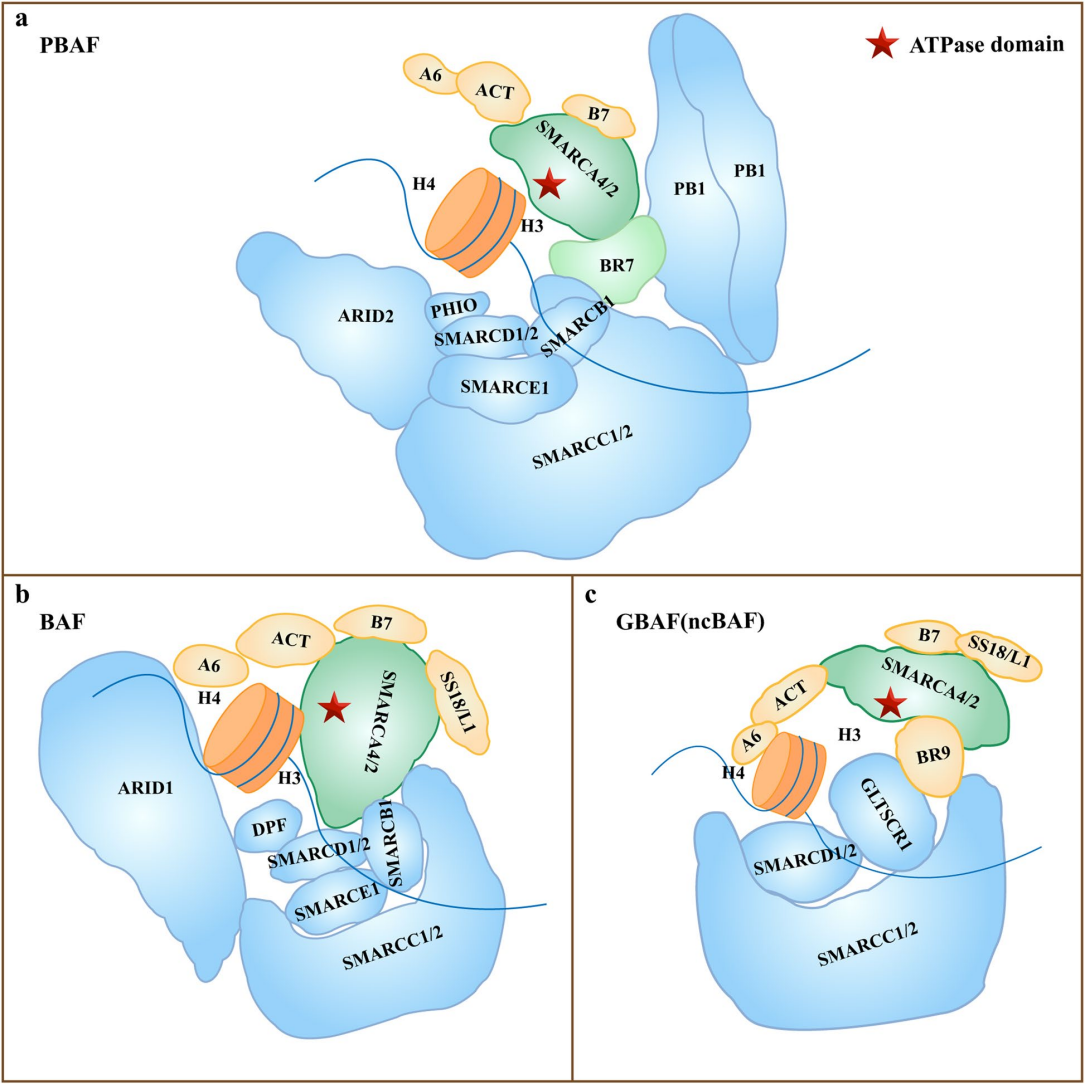
The structure of three SWI/SNF complexes There are three types of complexes: PBAF (**a**), BAF (**b**) and ncBAF/GBAF(**c**). The subunits of the SWI complex can be divided into motor module (green), substrate recruitment module (blue), and regulatory actin-related protein module (yellow) according to function. The ARID2, PBRM1,BAF45D, and BRD7 subunits are exclusive PBAF (**a**). GLTSCR1 subunit is unique to ncBAF/GBAF (**c**). The star is ATPase domain

The complex can be divided into three modules: the motor module, the substrate recruitment module (SRM) and the regulatory ARP module (actin-related proteins, ARPs). The motor module is the BRG1 (gene name SMARCA4) or BRM (gene name SMARCA2) subunit, which is the site where DNA translocation reactions occur. BRM and BRG1 have ATPase activity and can directly participate in chromatin remodeling by using ATP hydrolysis energy to reposition nucleosomes, exchange nucleosome histone dimers, or remove entire nucleosome octamers regulated by the ARP module [104–106]. The SRM encompasses a nucleosome-binding lobe (NBL), a DNA-binding lobe (DBL) and a histone tail (HBL). All three SWI/SNF complexes have an NBL and a DBL, while the HBL is unique to RSC and PBAF. The specific subunits in the SRM module are used to categorize SWI/SNF complexes into three types: BAF (BRG1 or BRM-related factor), PBAF (polybrominated related factor) and ncBAF/GBAF (GLTSCR1 or a complex containing GLTSCR1L and BRD9 [107, 108]. The SRM encompasses a nucleosome-binding lobe (NBL), a DNA-binding lobe (DBL) and a histone tail (HBL). All three SWI/SNF complexes have an NBL and a DBL, while the HBL is unique to RSC and PBAF [104, 109]. The purpose of the SRM is to adjust chromatin structure (via transcription factors and histone modifications) and target the motor module to regulate chromatin structure and gene transcription. Actin, ARP4, and BCL7A are the main components of the ARP module. These proteins function to connect the SRM and the motor module and regulate the activity of the motor module [110] (Fig. 2).

### Effect of SWI/SNF complex on glucose metabolism in tumors

The PBRM1 (PB1) subunit is a specific subunit of the PBAF SWI/SNF complex. Research has shown that approximately 46% of chronic kidney cell carcinomas have PBRM1 subunit mutations [111]. It has been found that in kidney cancer cells 786-O and SN12C, PBRM1 deficiency not only increases the levels of Akt and mTOR in the cells and their phosphorylated forms, activates AKT-mTOR signaling, but also increases the expression of mRNA and protein, which are key enzymes in glycolysis, such as phosphofructokinase (PFKP), enolase (ENO1), pyruvate kinase (PKM), and LDHA. Together with HIF1α, it promotes glycolysis of kidney cancer cells, and the researchers also observed that PBRM1 deficiency can promote the proliferation, migration and invasion of 786-O and SN12C cells, and high levels of PB1 can prolong the life expectancy of kidney cancer patients [112].

BRM is the ATPase subunit of the SWI/SNF complex. The expression of BRM, which is encoded by the SMARCA2 gene, is closely related to the expression of the glycolytic enzyme pyruvate kinase M2 (PKM2) and the AMPKα1-encoding gene (PRKAA1) in bladder cancer cells. The PRKAA1 gene encodes AMP-activated protein kinase (AMPK), and inactivation of AMPK promotes cellular metabolic reprogramming. BRM may be a negative regulator of PKM2 and AMPK-dependent metabolic processes in bladder cancer. Furthermore, BRM has different molecular characteristics in different bladder cancer cell lines. After the overexpression of BRM, the expression of almost all glycolytic genes (including PKM2), except fructose-1,6-bisphosphatase (FBP1), has been shown to increase in T24 bladder cancer cells, leading to increased glucose uptake. Compared to that in T24 bladder cancer cells, the expression of FBP1 is greater at both the protein and transcript levels in 5637 bladder cancer cells, resulting in decreased glucose uptake. The difference in FBP1 expression between the two cell lines may be the reason for the observed phenotypic differences. T24 bladder cancer cells are larger, spindle-shaped, and more basal-like, while 5637 bladder cancer cells are more tubular-like. Low expression of FBP1 is associated with the clinical stages of bladder cancer and is closely related to lower survival rates in bladder cancer patients (in whom there is more frequent urinary epithelial recurrence and metastasis) [113]. BRM plays an important role in glycolysis in bladder cancer cells by affecting the glycolytic process in bladder cancer cells through the influence of the key enzyme of glycolysis, FBP1, which in turn affects tumor phenotype. Differences in FBP1 expression also exist in different types of breast cancer; for example, basal-like breast cancer cells have a lower level of FBP1 expression than luminal breast cancer cells do, and FBP1 deficiency induces glycolysis and leads to an increase in glucose uptake, which is critical for EMT and basal-like breast cancer [114, 115].

The ARID1A gene is the most commonly mutated gene in 10%-15% of hepatocellular carcinomas (HCCs). Studies have shown that ARID1A deficiency leads to metabolic reprogramming in HCC cells by inhibiting the expression of the key glycolysis gene PKM. When glycolysis is suppressed, HCC cells shift their glucose metabolism toward the TCA cycle and oxidative phosphorylation. Additionally, HCC cells become more sensitive to copper, and the use of the copper ionophore elesclomol effectively treats ARID1A-deficient HCC in vivo. Researchers have proposed TCA cycle-targeted copper therapy as a promising and effective approach for treating ARID1A-deficient HCC patients [116].

**Fig. 3:**
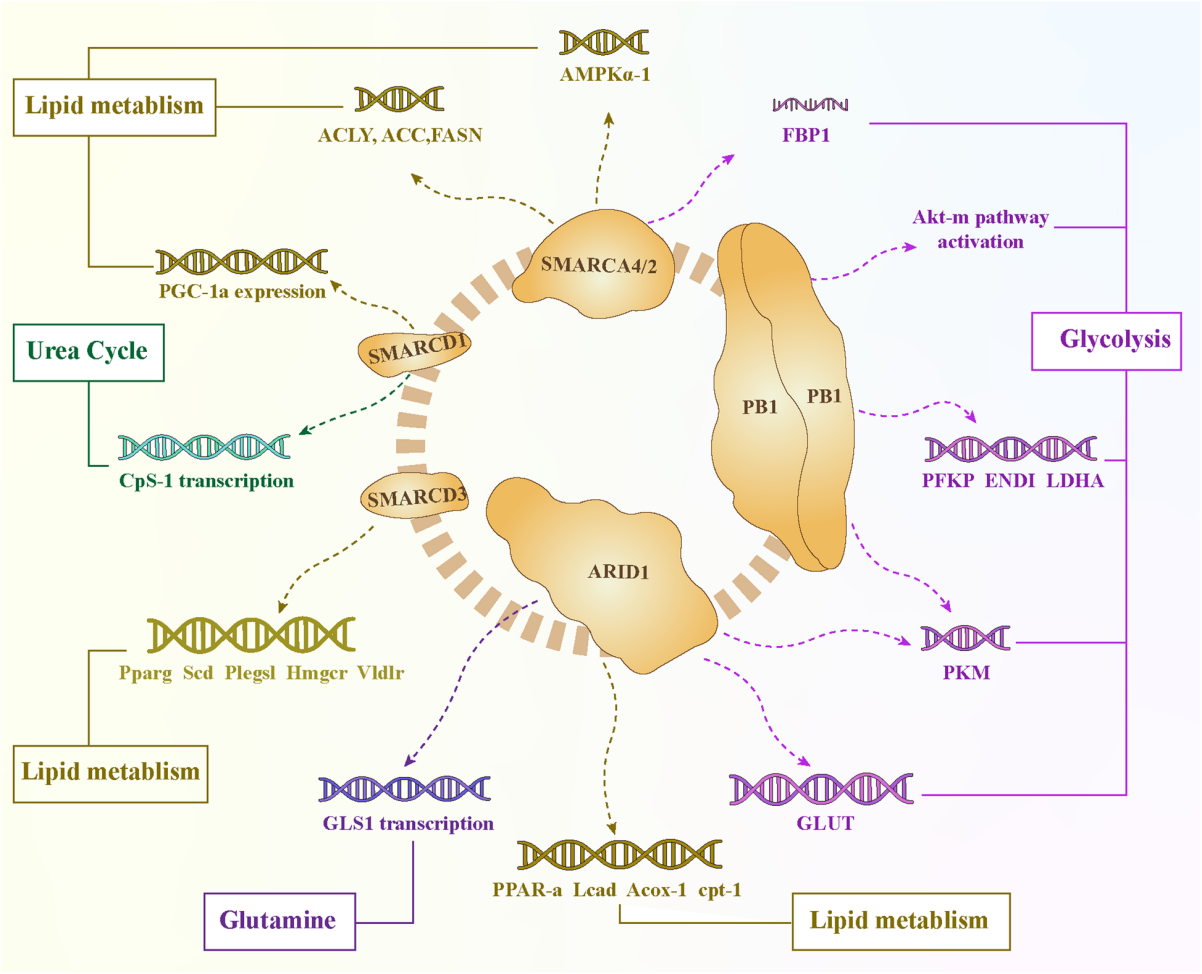
Schematic diagram of SWI/SNF complex regulating tumor metabolism The purple gene and arrow represent glucose metabolism. PB1 deficiency can increase the expression of PFKP, ENO1, PKM and LDHA, activate AKT-mTOR signaling, and significantly increase the concentration of lactate in cells. The coding gene SMARCA2 of BRM negatively regulates PKM2 to inhibit glycolysis, negatively regulates the expression of AMPKα1 coding gene PRKAA1 to inhibit the transport of glucose by GLUT-1, and overexpression of BRM can also increase the expression of FBP1 to promote cellular glycolysis. Deletion of ARID1A inhibits the expression of the key glycolysis gene PKM, leading to suppression of glycolysis. Cytoplasmic ARID1B interacts with c-RAF, stimulating the RAF-ERK signaling pathway. The activated transcription factors MYC and HIF1α then jointly promote the expression of GLUT and glycolytic enzymes, increasing glucose levels, glycolytic flux, and lactate production in cancer cells. Orange genes and arrows are associated with lipid metabolism. Silencing SMARCD1 can inhibit the activity and transcriptional activation ability of PGC-1α, downregulate the expression levels of fatty acid oxidation genes, and lead to significant accumulation of lipid droplets in liver cancer cells. SMARCD3 and FOXA1 cooperatively regulate the core genes involved in lipid and fatty acid metabolism, including Pparg, Scd1, Hmgcr, Ptgs1, and Vldlr, leading to an increase in the cellular content of fatty acids. Deletion of the ARID1A gene significantly reduces the expression of lipid beta-oxidation-related genes such as PPAR-alpha, Lcad, Acox-1, and cpt-1, leading to decreased lipid consumption and passive lipid accumulation. The blue gene and arrow represent glutamine metabolism. ARID1A is the first enzyme in glutamine breakdown and a transcriptional repressor of the rate-limiting enzyme GLS1, inhibiting glutamine metabolism through GLS1. The green gene and the arrow represent urea metabolism. Baf60a (SMARCD1) cooperates with Y-box binding protein-1 to inhibit the transcription of carbamoyl-phosphate synthetase 1 (CPS1), thereby suppressing urea synthesis

In addition, there is a potential link between the subcellular localization of the SWI/SNF complex subunit and glycolysis of tumor cells. The ARID1B (BAF250b) subunit is a typical nuclear tumor suppressor; however, ARID1B localized in the cytoplasm can interact with c-RAF (RAF1) and PPP1CA, thereby stimulating RAF-ERK signaling and β-catenin transcriptional activity. It is also associated with increased levels of ERK1 and ERK2 and the active form of β-catenin, which are closely related to the activation of the RAF-ERK signaling pathway and the Warburg effect. The activation of the RAF-ERK signaling pathway not only is closely related to the Warburg effect but also stimulates the expression of glucose transporters and glycolytic enzymes through the transcription factors MYC and HIF1α, thereby increasing glucose levels, glycolytic flux, and lactate production in cancer cells. Moreover, ERK can upregulate MYC expression through PKM2 phosphorylation to increase the phosphorylation of the glycolytic enzyme PFKFB2 and enhance glycolytic flux [117, 118] (Fig. 3).

### Effect of SWI/SNF complex on tumor lipid metabolism

Studies have shown that BAF60a is involved in the accumulation of cell lipid droplets and palmitic acid-induced cell senescence, in hepatoma cells HepG2, BAF60a (SMARCD1) is associated with PGC-1α activity and its transcriptional activation ability, FAO gene expression, when BAF60a (SMARCD1) is silenced, the expression of PGC-1α and FAO is down-regulated, and the lipid droplets of hepatocytes are significantly accumulated, on the contrary, when BAF60a is overexpressed, the expression of PGC-1α and FAO is up-regulated. Lipid droplet accumulation is reduced. Moreover, BAF60a overexpression can also inhibit palmitic acid-induced HepG2 cell senescence by inhibiting SA-β-Gal activity, p16/p21 expression, p-p38 expression, and γH2AX expression [119]. It has been found through ChIP-seq and RNA-seq that BAF60c (SMARCD3) is an epigenetic regulator of fatty acid metabolism, and its deletion will weaken fatty acid metabolism in vivo, and the study found that SMARCD3 co-regulate the transcription of lipid metabolism genes (arachidonic acid metabolism, fatty acid metabolism, cholesterol synthesis) with FOXA1, and several core genes of lipid metabolism Pparg, Scd1, Hmgcr, Ptgs1 and Vldlr are directly related to it Baf60c-BAF binds to FOXA1 and SMARCD3 loss down-regulates lipid transport, storage genes, and major transcriptional regulators of lipid metabolism. Tamoxifen-mediated SMARCD3 loss in tumor cells leads to a threefold decrease in the total free fatty acid content, which includes the monounsaturated fatty acid oleic acid, the polyunsaturated fatty acid docosahexaenoic acid, and the long-chain saturated fatty acid tridecanoic acid; these fatty acids are involved in the synthesis of complex lipids and play roles in cancer cell signaling and survival. The results of studies also suggest that the overexpression of Baf60c is sufficient to drive tumor growth in the absence of KRAS [120, 121].

Studies have shown that ARID1A is closely related to the expression of lipid β oxidation-related genes PPAR-α, Lcad, Acox-1, cpt-1 and other genes. When ARID1A is deficient, the chromatin accessibility of PPAR-α promoter and CPT1α is significantly reduced, and the expression of two key rate-limiting enzymes, CPT1α and ACOX1, is reduced for fatty acid oxidation. In this way, it can reduce lipid consumption, passively increases lipid accumulation, and promote the occurrence and development of liver cancer [122, 123].

### Effect of SWI/SNF complex on tumor alternative metabolic processes

The SWI/SNF complex can directly interact with the first enzyme and rate-limiting enzyme of glutamine metabolism, glutaminase 1 (GLS1) gene. Inactivation of the SWI/SNF complex increases the expression of GLS1. In a study of ovarian clear cell carcinoma (OCCC) cells, researchers mined published ChIP-seq data and found that SWI/SNF subunits such as ARID1A, SNF5, SMARCA4, and BAF155 can directly bind to GLS1. They further analyzed the relationship between the ARID1A subunit and OCCC glutamine metabolism and demonstrated that ARID1A is a transcriptional suppressor of GLS1. Knocking out ARID1A enhances the binding of RNA polymerase II to the GLS1 promoter. The glutaminase inhibitor CB-839 is effective at treating ARID1A-inactivated OCCC [124]. Furthermore, studies have shown that the SWI/SNF complex subunit Baf60a can participate in the maintenance of liver urea homeostasis. Baf60a, in conjunction with Y box-binding protein-1, inhibits the transcription of carbamoyl phosphate synthetase 1, thereby suppressing urea synthesis [125].

## Therapeutic strategies for tumor energy metabolism

Tumor metabolic fragility provides a novel way to develop therapeutic strategies, create personalized treatments, and tackle chemoresistance. Identifying the metabolic profile of tumor cells is crucial. For example, pancreatic cancer cells produce and release large amounts of lactate through glycolysis. This lactate inhibits immune cells in the tumor microenvironment and is linked to the cancer's high aggressiveness [126]. Experimental studies have demonstrated that targeting the glycolytic enzyme ENO1 can delay the progression of pancreatic cancer in mice and significantly extend survival [127]. Additionally, treatments that target metabolic pathways can be integrated with traditional chemotherapy, leading to the development of novel treatment strategies. In a recent study, drugs that target the SWI/SNF complex have effectively inhibited cancer cell growth, offering a promising new direction for tumor metabolism therapy [128].

### Therapeutic strategies targeting the glycolytic process

Inhibiting rate-limiting enzymes in glycolysis is a promising strategy for cancer treatment. The effects of rate-limiting enzyme inhibitors on tumor cells are generally similar: they primarily act on these enzymes to reduce glycolysis and ATP production, which induces apoptosis in the tumor cells. Recently, researchers have developed inhibitors that target key enzymes in the glycolytic pathway, including PKM2, and these have entered clinical trials. For instance, PKM2 is frequently overexpressed in several types of cancer, and inhibiting its activity can effectively slow tumor growth while enhancing sensitivity to chemotherapy. Studies indicate that PKM2 inhibitors enhance the response of tumor cells to targeted therapies. They also reduce drug resistance, which in turn improves patient prognosis [129]. Clinical trials have also explored the effects of other glycolysis inhibitors, such as lactate dehydrogenase A inhibitors and pyruvate dehydrogenase kinase inhibitors, which have shown promising anti-tumor activity in various cancer models, including non-small cell lung cancer (NSCLC) and HCC [130, 131].

While glycolytic inhibitors may theoretically reduce tumor growth, their clinical effectiveness is frequently inadequate. Many inhibitors face clinical limitations because they can be cytotoxic, leading to liver damage, cardiovascular issues, and immune system suppression. For instance, 2-deoxyglucose (2-DG) disrupts normal glycolysis by competitively inhibiting hexokinase (HK) and promotes apoptosis in various tumor cells [132, 133]. Dichloroacetic acid (DCA) is a DK inhibitor that specifically inhibits PDK1 in breast and prostate cancer cells. However, it is dependent on the cancer subtype and can be cytotoxic [134]. Drugs that block glucose metabolism in tumor cells can weaken tumor-infiltrating T cells, diminishing their effectiveness and aiding tumor growth [135]. Furthermore, when glycolysis is inhibited, tumor cells can adapt their metabolism to use alternative energy pathways, leading to less effective treatment outcomes [136].

Therefore, the development of novel inhibitors targeting specific molecules is particularly urgent. Researchers have discovered more drugs that have long half-lives, good solubility, and fewer side effects. These include chemotherapy drugs, targeted therapies, biologics, and natural products. For example, the HK oral inhibitor chlorinidamine (LND) selectively targets various tumors, and its adverse effects do not overlap when combined with other chemotherapy drugs. Consequently, LND is frequently utilized as a chemosensitizer to enhance the efficacy of chemotherapy drugs against tumors. Consequently, LND is frequently utilized as a chemosensitizer to enhance the efficacy of chemotherapy drugs against tumors [137]. Ikarugamycin (IKA) inhibits the glycolysis of pancreatic cancer cells by selectively binding to HK2, demonstrating a good anticancer effect without significant cytotoxicity in mice with pancreatic cancer xenograft models [138]. Gefitinib, a chemotherapy drug, slows the growth of NSCLC cells by disrupting their glycolysis process and inhibiting the PI3K-Akt-mTOR signaling pathway [139]. Biologics like the oncolytic adenovirus Ad-apoptin reduce glucose uptake and lactate production in NSCLC by blocking the AMPK/mTOR signaling pathway [140]. Several miRNAs target key glycolysis enzymes: miR-206 targets HK2, while miR-449a and miR-16-5p target LDHA [141–143]. Overexpression of miR-603 reduces glucose uptake and lactate production, and it also inhibits the proliferation, migration, and invasion of ovarian cancer cells by targeting HK2 [144]. miR-29b negatively regulates the expression of AKT2 and AKT3, downregulates HK2 and PKM2, and reduces the Warburg effect, thereby slowing ovarian cancer progression [145]. Natural products like Shikonin, which binds to and inhibits pyruvate kinase (PK) while regulating glycolysis in LAUD [146]. The plant-derived flavonoid apigenin reduces GLUT1 and GLUT3 levels, decreases glucose metabolic activity, and increases apoptosis in thyroid cancer cells [147]. Chrysin, a natural flavonoid, inhibits glycolysis and promotes apoptosis in HCC cells by downregulating HK-2 expression [148]. Oleuropein, an iridoid ether terpene glycoside found in plants, targets glucose-6-phosphate isomerase (GPI) to inhibit glycolysis and tumor growth in liver cancer [149]. Bendicycloheptene sulfonate (OBHS) inhibits glycolysis in breast cancer cells by downregulating GLUT1 expression, thus slowing cancer progression and proliferation [150].

### Therapeutic strategies targeting mitochondrial metabolism

Therapeutic strategies targeting mitochondrial metabolism are also gaining traction. TCA cycle enzyme inhibitors, such as the mutant isocitrate dehydrogenase (IDH) inhibitors AG-221 and AG-881, are already in clinical trials for the treatment of IDH2 or IDH1/2-mutated acute myeloid leukemia [151, 152]. CPI-613, which targets the α-ketoglutarate dehydrogenase complex and pyruvate dehydrogenase, is being investigated for the treatment of leukemia, lymphoma, and small cell lung cancer [153, 154].

Oxidative phosphorylation inhibitors, including mitochondrial complex I (NADH) inhibitors and metformin, are currently in clinical trials for treating breast, colorectal, and prostate cancers [155, 156]. IACS-010759 is being investigated for the treatment of brain cancer and acute myeloid leukemia, and it has been shown to effectively reduce the growth rate of tumor cells [157]. Additionally, atovaquone has been shown to inhibit cancer cell proliferation by targeting complex III. It also exhibits antitumor activity in animal models of acute myeloid leukemia, acute lymphoblastic leukemia, cervical cancer, breast cancer, and gastric cancer [158–161]. Doxorubicin and photofrin sodium, which is approved for esophageal cancer and non-small cell lung cancer, can inhibit complex IV [162]. The complex V inhibitor niclosamide is in phase I/II clinical trials for prostate and colon cancer, and nitazoxanide is in phase II clinical trials for different types of advanced cancer [163].

Etomoxir, an inhibitor that targets CPT1, stops tumor progression in a model of high-grade serous ovarian cancer during fatty acid oxidation (FAO) [164]. However, etomoxir is toxic. Excessive etomoxir can promote macrophage polarization and inhibit T cell differentiation, resulting in anti-tumor immune-related off-target effects [165, 166]. In contrast, inhibitors of other enzymes in FAO, such as trimetazidine and ranolazine, which inhibit 3-ketoyl-CoA thiolase, were not observed in cell lines, primary cells, or mice [167]. There is a need to develop FAO-targeted inhibitors that possess high specificity and low toxicity.

In glutamine metabolism, the GLS inhibitor CB-839 has entered clinical trials for treating hematologic malignancies, colorectal cancer, melanoma, NSCLC, TNBC, and others [168]. Studies have shown that R162, an inhibitor of high glutamate dehydrogenase (GDH), can inhibit the growth of glioma by causing an imbalance in redox homeostasis [169].

Additionally, mitochondrial dynamics, including fusion and fission processes, are closely linked to tumor resistance [170]. Chemotherapy-resistant ovarian cancer cells contain numerous interconnected mitochondria [171]. Strategies targeting mitochondrial dynamics, such as intervening in the processes of mitochondrial fusion and fission, are considered potential therapeutics. GTPase dynein-associated protein 1 (DRP1) is crucial for mitochondrial fission. Its inhibitor, Mdivi-1, can reduce mitochondrial fission, lower oxidative metabolism in colorectal cancer cells, and impede cell proliferation [172]. Atractylolactone I can block DRP1-mediated mitochondrial fission and disrupt mitochondrial membrane integrity, thereby inducing apoptosis [173]. Bromodomain inhibitors (BETi) downregulate the proteins involved in mitochondrial fission in triple-negative breast cancer cells and increase the expression of fusion mitochondria, which impairs mitochondrial fission, affects OXPHOS, and promotes cell death [174]. Research indicates that knocking down ephrin type A receptor 2 (EPHA2) enhances mitochondrial fusion, reduces mitochondrial fission, and promotes mitophagy and autophagy. Additionally, the drug sesamol targets EPHA2 to improve cisplatin sensitivity in cervical cancer by regulating mitochondrial dynamics [175]. The androgen receptor antagonist apalutamide (ARN) and the complex I inhibitor IACS-010759 (IACS) regulate the process of mitochondrial fission and fusion in prostate cancer, and the combination of the two can increase androgen-sensitive prostate cancer cell death and mitochondrial oxidative stress [176]. Moreover, several naturally derived compounds, including inauhzin, matrine, aloe vera gel glucomannan, as well as chemotherapy drugs like triptolide and camptothecin, influence mitochondrial dynamics by stimulating DRP1 phosphorylation and dephosphorylation, showing promise for colorectal cancer treatment and clinical application [177] (Table 1).

**Table 1:** Therapeutic targeting of metabolic pathways

**Metabolism**	**Tagrgets**	**Inhibits**	**Ref**
Glycolysis	HK	2-DG	[132, 133]
	HK	LND	[137]
	HK	IKA	[133]
	HK	miR-206	[141]
	HK	miR-603	[138]
	HK	Chrysin	[148]
	GPI	Oleuropein	[149]
	LDHA	miR-449a	[142]
	LDHA	miR-16-5p	[143]
	PDK1	DCA	[134]
	PK	Shikonin	[146]
	GLUT-1	BAY-876	[15]
	GLUT-1	OBHS	[150]
	GLUT-1/3	Apigenin	[147]
	PI3K-Akt-mTOR	Ad-apoptin	[140]
	PI3K-Akt-mTOR	miR-29b	[145]
Mitochondrial metabolism	IDH	AG-221, AG-881	[151, 152]
	OGDC	CPI-613	[153, 154]
	PDH	CPI-613	
	Respiratory chain complexI	metformin	[155, 156]
	Respiratory chain complex I	IACS-010759	[157]
	Respiratory chain complex I	IACS-010759	[158]
	Respiratory chain complex III	Atovaquone	[158–161]
	Respiratory chain complex IV	Doxorubicin, Photofrin	[162]
	Respiratory chain complex V	Niclosamide, Nitazoxanide	[163]
Fatty acid oxidation	CPT1	etomoxir	[164–166]
	DRP1	Mdivi-1	[172]
	DRP1	Atractylenolide I	[173]
Glutamine metabolism	GLS	CB-839	[124]
	GDH	R162	[169]

### Exploiting the metabolic vulnerabilities of tumors

Metabolic vulnerability describes how tumor cells rely on specific metabolic pathways, creating new opportunities for targeted therapy [178, 179]. Researchers can develop therapeutic strategies by identifying cancer cells’ dependence on specific nutrients or metabolic pathways. For example, targeted therapy for glutamine metabolism can effectively reduce the proliferation of acute myeloid leukemia cells by blocking glutamine uptake [180]. Dependence on the mTOR pathway often causes drug resistance to targeted therapies; thus, targeting this pathway may help overcome resistance to drugs like PI3K inhibitors (alpelisib and pictilisib) [181]. In imatinib-resistant gastrointestinal stromal tumors (GIST), the GIST 882 cell line exhibits elevated glycolysis and OXPHOS levels, making it more susceptible to glycolysis inhibition, while the GIST-T1 cell line shows reduced mitochondrial respiration and is more vulnerable to OXPHOS inhibition [182]. Many tumors depend heavily on fatty acid synthesis. Acetyl-CoA carboxylase 1 (ACC1), the rate-limiting enzyme in this process, has emerged as a target for treating acute myeloid leukemia, breast cancer, ovarian cancer, non-small cell lung cancer, and liver cancer [183, 184]. AG-120, a mutant isocitrate dehydrogenase (IDH)1 inhibitor, has been approved by the United States Food and Drug Administration as a first-line treatment for IDH1-mutant acute myeloid leukemia [185]. These targeted therapies can be used alone or in combination with other treatments to enhance their effectiveness.

Synthetic lethality is a method that selectively kills tumor cells while sparing normal cells by simultaneously blocking two or more interdependent metabolic pathways. For example, the GLS1 inhibitor BPTES works synergistically with olaparib to inhibit the growth of VHL-deficient renal cell carcinoma (RCCC) [186]. CB-839, a small molecule inhibitor of GLS1, is used in combination with erlotinib to inhibit the growth of EGFR-mutated NSCLC and with Glutor, an inhibitor of the glucose transporter GLUT1/2/3, to suppress colon cancer cell growth [187, 188]. Milk thistle silibinin directly interacts with the GLUT4 subtype to inhibit glucose uptake. When combined with paclitaxel, it significantly reduces ovarian cancer cell growth, proving more effective than paclitaxel alone [189]. The ATP citrate lyase (ACLY) inhibitor SB-204990, in combination with glutamine deprivation, causes KRAS-driven cancer cell death [190]. Tumors deficient in SMARCA4/2 depend on OXPHOS for survival and are highly sensitive to inhibitors targeting either OXPHOS or glutamine metabolism. At clinically relevant doses, alanine supplementation has a synergistic effect with OXPHOS inhibition or conventional chemotherapy, leading to a significant reduction in tumor activity [191]. Combining inhibitors that target multiple metabolic pathways can enhance therapeutic efficacy. For instance, when the mitochondrial complex I and fission inhibitor MDIVI-1 is used alone, breast cancer cells maintain their energy requirements for growth by enhancing glycolysis. However, when combined with the glycolysis inhibitor 2DG, breast cancer cell death is significantly increased [192]. OXPHOS metabolism inhibitors, when used in combination with glucose metabolism inhibitors, such as Marizomib (a dual inhibitor of proteasomes and OXPHOS), synergistically inhibit the activity of triple-negative breast cancer alongside the glycolysis inhibitor 2DG [193]. Studies have shown that the combination of mitochondrial complex III inhibitors resveratrol and tamoxifen can overcome tamoxifen resistance in breast cancer cells [194].

"Metabolic checkpoints" refer to important enzymes or receptors in metabolic pathways whose activity levels can affect immune cell function [195]. Tumor cell metabolism alters the microenvironment, making it acidic and hypoxic. In this environment, tumor cells compete with immune cells for nutrients like glutamine, which impacts immune cell function and the efficacy of immunotherapy. Accumulation of lactate and amino acid metabolites in the tumor microenvironment inhibits T cell activation and promotes the survival of Treg cells [196]. Lactate plays a key role in transforming macrophages into tumor-associated macrophages (TAMs) [197]. Most TAMs are considered the M2 phenotype, which is anti-inflammatory and pro-tumor [198]. High lactate concentrations hinder NK cell immune surveillance, allowing tumor cells to evade attack by the immune system [199]. Sodium chloride in the tumor microenvironment can enhance T cell metabolic adaptation, thereby enhancing the activation status and effector function of CD8 T cells [200]. Modulating the various metabolic functions of immune cells is another important branch of immunotherapy [201].

Indoleamine 2,3-dioxygenase (IDO) inhibitors are a type of immunotherapy drug developed based on "metabolic checkpoints." These inhibitors can block tryptophan metabolism in cancer cells and activate T cells [202]. Initially, the IDO inhibitor Epacadostat, when combined with Keytruda (a PD-1 antibody), failed in a phase III clinical trial for malignant melanoma. This failure was due to increased side effects and did not extend the patients' progression-free survival (PFS) or overall survival (OS) [203]. In the most recent clinical trial, the combination of IDO inhibitors with chemotherapy and radiotherapy was successfully tested in phase I for pediatric brain tumors and is expected to enter phase II/III trials soon [204]. Additionally, studies indicate that quinolineate, a product of tryptophan metabolism, serves as a metabolic checkpoint in glioblastoma. It does this by inducing NMDA receptor activation and activating Foxo1/PPARγ signaling in macrophages [205].

Avasimibe, an inhibitor of acetyl-CoA acetyltransferase 1 (ACAT1), has demonstrated significant anti-tumor effects in a mouse model of melanoma. Additionally, combining avasimibe with an anti-PD-1 antibody has improved anti-tumor immunotherapy[206]. Glutamine metabolism serves as another potential "metabolic checkpoint" in cancer immunotherapy. Glutamine inhibitors can reduce tumor cell activity, while activated CD8T cells can adapt to glutamine blockade by increasing acetate metabolism [195]. The glutamine antagonist 6-diazo 5-oxo-l-norleucine (DON) inhibits various glutamine-utilizing enzymes and reduces tumor cell viability, but it also has toxic side effects. To address this, Leone et al. developed a novel DON prodrug that activates only in the tumor microenvironment, thereby avoiding toxic off-target effects on other tissues [207]. Zheng et al. developed a hypoxia-activated prodrug, HDON, which, when combined with combretastatin A4 nanoparticles, selectively kills tumor cells in animal models and significantly increases tumor suppression rates [208]. Research has shown that glucose-6-phosphate dehydrogenase (G6PD) serves as a metabolic checkpoint in tumor-activated cytotoxic T lymphocytes. It regulates the expression of granzyme B, which is positively correlated with the response of mesothelioma patients to immune checkpoint inhibitor immunotherapy [209]. G6PD also serves as a critical metabolic checkpoint for the metabolism and functional reprogramming of regulatory T cells [210]. Therefore, an in-depth study of the metabolic characteristics of tumor cells and their relationship with immune responses can enhance the overall effectiveness of tumor therapies. Additionally, this research is significant for developing new targeted therapies that enable more precise and individualized treatment.

### Recent advances in the use of SWI/SNF complexes in targeted drug therapy

The SWI/SNF complex plays a crucial role in tumor metabolism, making it an attractive target for therapy. Currently, clinical studies are evaluating drugs that target this complex to assess their potential in various cancer types, which could lead to new therapeutic strategies. The POU2F3-POU2AF2/3 transcription factor complex is a key regulator of small cell lung cancer (SCLC). The POU2F3 subtype (SCLC-P) relies specifically on the activity of the mSWI/SNF chromatin remodeling complex. Notably, orally available mSWI/SNF ATP enzyme degraders significantly inhibit tumor growth in preclinical models of SCLC-P and multiple myeloma without causing toxicity [211]. Furthermore, recent studies indicate that the SWI/SNF complex influences the effectiveness of targeted therapies by regulating tumor lipid metabolism. In patients with advanced LAUD harboring mEGFR mutations, lipid metabolism and co-mutations in genes such as ARID1A, ARID1B, BCR, and RBM10 are linked to the efficacy of icotinib (an epidermal growth factor receptor-tyrosine kinase inhibitor, EGFR-TKI). These four co-mutated genes are significantly associated with shorter progression-free survival. Additionally, these findings are closely related to the glycerophospholipid and sphingolipid metabolism pathways [212].

## Clinical significance and translational potential, challenges, and future directions

Research on metabolism-related targets has led to new therapeutic strategies, particularly for cancer and metabolic diseases. Drugs that target specific metabolic enzymes and metabolites, like the glycolytic pathway, have been clinically used for tumor imaging and treatment. An example is difluorodeoxyglucose (FDG) [213]. Additionally, advancements in nanomedicine offer innovative approaches to drug delivery systems, increasing drug concentration in tumor tissues while minimizing damage to normal tissues [214]. Furthermore, researchers can quickly identify new compounds with potential therapeutic effects using computational chemistry and high-throughput screening techniques, which accelerates the drug development process.

### Overcoming drug resistance in metabolically targeted therapies

Tumor cells survive and proliferate due to metabolic reprogramming; however, this adaptation also results in resistance to therapies that target metabolism. Drug resistance can occur through several mechanisms, such as changes in metabolic pathways, increased drug efflux pump expression, altered intracellular signaling, and modifications to the tumor microenvironment [215]. For instance, melanoma cells with the BRAF V600 mutation develop resistance to BRAF inhibitors through metabolic reprogramming after undergoing targeted therapy [216]. Changes in lipid metabolism significantly contribute to resistance against anti-tumor therapies. Tumor cells can improve their survival by regulating lipid synthesis and degradation, which helps them resist drug treatments [217].

Researchers have proposed several strategies to combat these resistance mechanisms. These include combining drugs that work through different mechanisms and reversing resistance by targeting key enzymes in metabolic pathways [218]. For example, inhibiting targeted purine metabolism significantly improves glioblastoma's radiosensitivity [219]. Disrupting mitochondrial metabolism in rectal cancer cells can inhibit their growth and increase their sensitivity to chemotherapy [220]. Combining glutamine inhibitors with prostate chemotherapy can enhance treatment efficacy [221]. Blocking glutamine metabolism in EGFR-mutated non-small cell lung cancer significantly increases sensitivity to almonertinib [222]. The fatty acid transporter CD36 plays a crucial role in lapatinib resistance in breast cancer; inhibiting CD36 expression reduces fatty acid uptake by lapatinib-resistant breast cancer cells [223].

### Metabolic therapy combined with other therapies

The combination of metabolic therapy with other treatment modalities has become an important strategy to improve the efficacy of cancer treatment. Research indicates that combining metabolic inhibitors with immune checkpoint inhibitors can greatly improve the anti-tumor immune response. For example, therapies targeting glucose or glutamine metabolism are combined with PD-1/PD-L1 checkpoint inhibitors [224]. Moreover, metabolic interventions can enhance the effectiveness of chemotherapy and radiotherapy. They also increase the sensitivity of tumor cells to these treatments [225]. For instance, HK2-mediated glycolysis influences the efficacy of crizotinib, an ALK inhibitor. Additionally, 2DG enhances the sensitivity of non-small cell lung cancer to crizotinib by inhibiting both HK2-mediated glycolysis and the AKT/mTOR signaling pathway [226]. Knocking down the GLUT3 gene in combination with radiotherapy can significantly inhibit glioblastoma proliferation and help address radiation resistance [227].

Recently, advancements in nanobiomaterials have led to the development of various nanomaterials, including organic, inorganic, lipid, polysaccharide compounds, and synthetic polymers. These nanomaterial-based drug delivery systems enhance the efficacy of therapies like photothermal therapy, photodynamic therapy, radiotherapy, chemotherapy, and immunotherapy [228, 229]. Nanomedicine that targets mitochondrial metabolism offers a promising therapeutic approach [230]. Studies have shown that 3-O-β-D-galactosylated resveratrol polydopamine nanoparticles significantly inhibit tumor growth in liver cancer model mice without causing noticeable toxicity to major organs [231].

However, combining metabolic therapy with other treatments comes with challenges. These include metabolic adaptation and the complexity of the microenvironment [232]. Therefore, future research should focus on optimizing the timing and dosage of combination therapies to enhance synergies and improve treatment efficacy.

### Innovations in targeted tumor metabolism

In recent years, the rise of single-cell metabolomics has enabled researchers to analyze the metabolic characteristics of tumor cells at the single-cell level, revealing the metabolic heterogeneity of different tumor cell subsets [233]. This lays the groundwork for improved diagnosis, patient classification, personalized treatment, and targeting specific metabolic pathways and cell types [234]. Single-cell and spatial protein transcriptomics have identified RE1 silencing factor (REST) as a potential biomarker for small cell lung cancer, associated with low neuroendocrine features, increased anti-tumor immunity, and prolonged survival [235]. Additionally, single-cell sequencing technology has shown that the expression of the metabolic enzyme 6-phosphogluconate dehydrogenase (PGD) is significantly up-regulated in LUAD. PGD promotes glycolysis and fatty acid synthesis in LUAD cells by reducing p-AMPK levels, suggesting that PGD could serve as a prognostic biomarker and therapeutic target for LUAD [236]. Metabolomics can identify specific metabolites resulting from the metabolic reprogramming of tumor cells, which may act as potential biomarkers for early cancer detection and monitoring. For instance, clear cell renal cell carcinoma exhibits higher levels of glutamine compared to normal kidney cells [237]. A biomarker panel for the diagnosis of early lung adenocarcinoma was identified through metabolomics of serum from lung adenocarcinoma patients, with a sensitivity of 70%-90% and a specificity of 90%-93%, including seven metabolites (histamine, cysteine, fatty acids (18:2), uracil, uric acid, 3-hydroxypicolinic acid (HPA), indoleacrylic acid (IA)) and related pathways [238]. Liquid biopsy techniques can detect tumor-specific metabolites through blood samples [239]. For example, a specific combination of metabolites in the serum of patients with hepatocellular carcinoma can distinguish between early and advanced tumors [240].

Furthermore, advancements in Deuterium Metabolic Imaging (DMI) technology enable real-time monitoring of tumor metabolic status, offering a novel method for diagnostic and therapeutic monitoring in clinical practice. For instance, 18F-FDG PET imaging technology allows for the assessment of glucose metabolism in tumor cells and can predict patient responses to immunotherapy [241]. DMI can dynamically track the entire cellular metabolism process in tumor cells, including glucose transport, the pentose phosphate pathway, glycolysis, and citric acid cycling, through exogenous ^2^H labeling. This capability helps identify the optimal time window for immunotherapy and enhances the precision of clinical interventions [242, 243]. Another type of DMI can quantify key metabolites in oncology, such as glucose, lactate, glutamate, and glutamine, to assist in diagnostic analysis, treatment response monitoring, and the identification of new therapeutic targets for various cancer subtypes [244]. Future research should integrate advanced metabolomics and imaging technologies to investigate the mechanisms of metabolic reprogramming, identify new targets, and facilitate the clinical translation of metabolic targeted therapies for tumors.

The influence of SWI/SNF complex in drug resistance has also been gradually emphasized. Research has shown that the combined use of SWI/SNF complex subunit-related inhibitors during tumor treatment can enhance the sensitivity of tumors to drugs. The stable knockdown of the SWI/SNF catalytic subunits BRG1 and BRM in lung cancer and head/neck cancer cells can enhance the sensitivity of cells to cisplatin because the downregulation of BRG1 and BRM hinders the repair of DNA intrastrand adducts and interstrand crosslinks (ICLs) [245]. In the ovary, high expression of SMARCA2 affects the expression of cell cycle- and apoptosis-related proteins in ovarian cancer cells, increasing their resistance to cisplatin [246]. According to the latest research, low SMARCA4 expression and SMARCA2 overexpression are associated with platinum resistance in high-grade serous ovarian cancer (HGSC) cells, possibly related to substantial changes in chromatin accessibility, the activation of fibroblast growth factor (FGF) signaling, the activation of the MAPK pathway, and reduced apoptosis [247]. Research has shown that the glutamate-cysteine ligase catalytic subunit (GCLC) could serve as a new therapeutic target for ARID1A-deficient ovarian cancer cells. BLC7A11 expression is impaired in ARID1A-deficient cancer cells, leading to decreased cellular levels of the antioxidant glutathione (GSH). As a result, these cells are more sensitive to GSH and GCLC [248]. In pancreatic cancer, pancreatic cells with SWI/SNF dysfunction exhibit significantly increased sensitivity to DNA-damaging agents, particularly DNA cross-linking agents (such as cisplatin and oxaliplatin) [249]. However, the knockout of the ARID1A subunit of the SWI/SNF complex can lead to gemcitabine resistance in pancreatic cancer cells. Mechanistically, ARID1A directly binds to the promoter of RRM2 (a gemcitabine resistance-related gene), and the knockout of ARID1A reduces the transcription of RRM2 [250]. In breast cancer, the knockout of the ATPase subunit SMARCA4 of the SWI/SNF chromatin remodeling complex is associated with cisplatin resistance in triple-negative breast cancer cells. After SMARCA4 knockout, the epithelial–mesenchymal transition (EMT) pathway and Hippo-YAP/TAZ target genes are activated in cells. The YAP1 inhibitor verteporfin can reduce the viability and invasiveness of SMARCA4-knockout cells and may serve as a new therapeutic strategy for cisplatin-resistant patients [251]. Therefore, in-depth understanding of the mechanism of drug resistance and the development of corresponding countermeasures will be an important direction for future research (Table 2).

**Table 2:** The SWI/SNF complex submits targets in tumor therapy

**Cancer**	**Submits**	**Antineoplastic drugs**	**Outcome**	**Ref**
Lung adenocarcinoma with mEGFR mutations	ARID1A, ARID1B, BCR, BM10	Icotinib	Potential predictive value of serum targeted metabolites and concurrently mutated genes for EGFR-TKI therapeutic efficacy in lung adenocarcinoma patients with EGFR sensitizing mutations	[212]
Lung cancer, head/neck cancer	BRG1, BRM	Cisplatin	Downregulation of SWI/SNF chromatin remodeling factor subunits modulates cisplatin cytotoxicity	[245]
Ovarian cancer	SMARCA2/4	Cisplatin	Overexpression of SMARCA2 or CAMK2D is associated with cisplatin resistance in human epithelial ovarian cancer	[246]
			Ovarian high-grade serous carcinoma cells with low SMARCA4 expression and high SMARCA2 expression contribute to platinum resistance	[244]
	ARID1A	GSH, GCLC	Targeting the Vulnerability of Glutathione Metabolism in ARID1A-Deficient Cancers	[248]
Pancreatic cancer	ARID1A	Cisplatin, Oxaliplatin, Gemcitabine	ARID1A promotes chemosensitivity to gemcitabine in pancreatic cancer through epigenetic silencing of RRM2	[249]
Breast Cancer	SMARCA4	Cisplatin	SMARCA4 Depletion Induces Cisplatin Resistance by Activating YAP1-Mediated Epithelial-to-Mesenchymal Transition in Triple-Negative Breast Cancer	[251]

## Conclusion

This review examines the key pathways of tumor energy metabolism and their regulatory mechanisms, focusing on glycolysis and mitochondrial oxidative phosphorylation. Identifying key enzymes offers clear targets for drug development and establishes a foundation for new therapeutic strategies. Currently, many new drugs targeting tumor-metabolizing enzymes have issues like low specificity, immune cell suppression, and side effects. In contrast, natural products from various plants show better specificity and fewer side effects, offering new avenues for developing key metabolic enzyme inhibitors. Additionally, we discuss the crucial role of the SWI/SNF complex in tumor energy metabolism. Studies show that the SWI/SNF complex significantly impacts the metabolic pathways of tumor cells by regulating gene expression and chromatin remodeling. Therapeutic strategies targeting the SWI/SNF complex and its downstream metabolic pathways are expected to yield new breakthroughs in tumor treatment. Future research must prioritize the role of SWI/SNF complexes in various tumor types and their microenvironments to gain a comprehensive understanding of their function in tumor energy metabolism.

As clinical trials advance, targeted therapies for metabolism show great promise. Combining metabolic targeted therapy with immunotherapy and chemotherapy will be crucial for developing cancer treatments and may create new opportunities. Meanwhile, as personalized medicine grows, utilizing advanced technologies like metabolomics will become a key research area. High-throughput transcriptomics, metabolomics, and single-cell sequencing technologies will enhance our understanding of tumor metabolism. This knowledge will help us identify new therapeutic targets and biomarkers and optimize treatment options for individualized therapies.

## Data Availability

Not applicable.
